# Tuning by Hydrogen Bonding in Photosynthesis

**DOI:** 10.1021/acs.jpcb.4c04405

**Published:** 2024-09-18

**Authors:** Kõu Timpmann, Margus Rätsep, Erko Jalviste, Arvi Freiberg

**Affiliations:** † Institute of Physics, University of Tartu, W. Ostwaldi 1, 50411 Tartu, Estonia; ‡ Estonian Academy of Sciences, Kohtu 6, 10130 Tallinn, Estonia

## Abstract

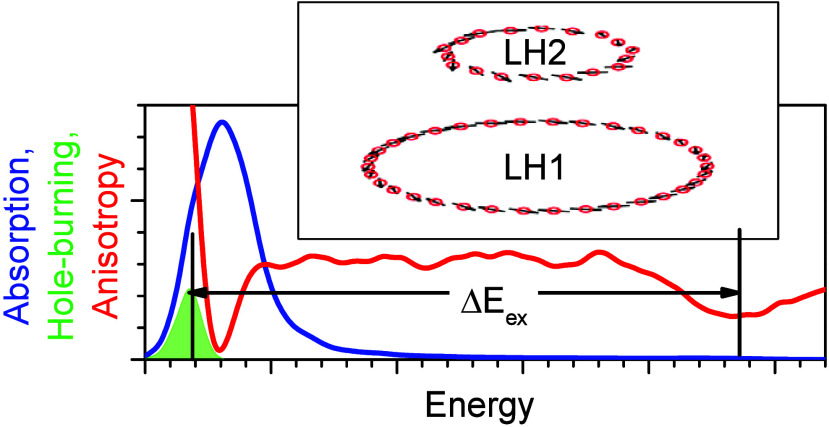

Hydrogen bonding plays a crucial role in stabilizing proteins throughout their folding process. In photosynthetic light-harvesting chromoproteins, enriched with pigment chromophores, hydrogen bonds also fine-tune optical absorption to align with the solar irradiation spectrum. Despite its significance for photosynthesis, the precise mechanism of spectral tuning through hydrogen bonding remains inadequately understood. This study investigates wild-type and genetically engineered LH2 and LH1 light-harvesting complexes from *Rhodobacter sphaeroides* using a unique set of advanced spectroscopic techniques combined with simple exciton modeling. Our findings reveal an intricate interplay between exciton and site energy shift mechanisms, challenging the prevailing belief that spectral changes observed in these complexes upon the modification of tertiary structure hydrogen bonds almost directly follow shifting site energies. These deeper insights into natural adaptation processes hold great promise for advancing sustainable solar energy conversion technologies.

## 1 Introduction

Hydrogen bonding (H-bonding) plays a critical role in stabilizing proteins during various stages of folding. In chromoproteins found in photosynthetic light-harvesting systems, where pigment chromophores are abundant, the H-bonds formed between the pigments and the surrounding protein matrix serve several purposes. Not only do they contribute to the overall stability of the protein physical structure, but they also suppress static and dynamic disorders in the electronic structure of closely packed pigment aggregates, while fine-tuning their collective absorption properties to optimize collection of the solar irradiation spectrum.^1^

Despite the widespread significance of H-bonding in this context, which enables plants, algae, and phototrophic bacteria to flourish in their respective ecological niches, the precise mechanism by which it influences color tuning remains to be better understood. This is because a great variety of factors apart from H-bonding may be considered responsible for spectral tuning of photosynthetic chromophores such as protein electrostatics,^2,3^ macrocycle ring deformation,^3−6^ orientation and dynamics of macrocycle functional groups,^7^ and axial ligation.^4,8^ Furthermore, close proximity of the pigment chromophores in light-harvesting complexes required for efficient energy transfer supports collective (delocalized) excited states called excitons with shifted spectra with respect to individual pigments.^9^ Within the most basic exciton model,^10,11^ the tuning of light-harvesting spectra can be understood as a combined effect of the interactions between transition densities of the pigments (an exciton shift), and between the pigments and the residues in their protein binding pockets (a site energy shift), the two shift components naively thought to be independent from one other. Static and dynamic variations of the chromoprotein conformations necessarily reflect in both the exciton site energies and couplings, shifting and broadening individual exciton states, and increasing the spread (bandwidth) of the exciton state manifold.^12,13^ The disorders generally also facilitate abrupt localization (self-trapping) of light-harvesting excitons,^14−17^ reviewed in ref (18).

While the concept of photosynthetic excitons is generally acknowledged,^9^ their effectiveness in elucidating spectral shifts in light harvesting has encountered constraints in both theoretical and experimental contexts. These limitations can be attributed to two primary factors. First, although significant progress has been made in recent years in the atomistic modeling of exciton states of light-harvesting complexes,^11,19−21^ ongoing research continues to focus on accurately accounting nonadiabatic vibronic coupling effects and understanding the role of mixing with higher energy states.^22−27^ Second, the scarcity of native photosystems suitable for straightforward experimental evaluations hinders the distinct separation of site energy and exciton shift mechanisms, preventing a more in-depth understanding of the phenomenon. Consequently, with a few notable exceptions,^1,28−34^ the observed spectral shifts related to adjustments of the H-bonding pattern in light-harvesting chromoproteins have largely been assigned to just site energy shifts.^3−5,35−39^ However, efforts of quantitative evaluations of the shifts due to individual mechanisms have provided incompatible results. For example, energetic effects of H-bonding between C3 acetyl carbonyl and surrounding protein residues in related chromoprotein complexes vary between about 90 and 500 cm^–1^.^28,30−32^

A couple of recent structural studies well illustrate the ambivalence of this situation. In ref (34) the heptameric B800–828 complex from *Marichromatium purpuratum* was investigated, and it was found that the B828 pairs of bacteriochlorophyll *a* (BChl) pigments were H-bonded to C-terminal residues on the α and β polypeptides. Examination of the spacings between the 14 BChl pigments in the B828 ring showed significantly increased separation between B828 dimers relative to a B800–850 complex and that the 7-mer ring imposes an increased angle between dimers. It was suggested that these two factors lower the strength of dimer–dimer exciton coupling, which causes the blueshift of the B850 band to 828 nm. Thus, H-bonding did not appear to play a role in shifting the Q_*y*_ absorption in the heptameric LH2. Yet a subsequent quantum chemical elucidation^40^ arrived at a different conclusion that the spectral properties of this complex are tuned by site energies along with exciton couplings. In another structural work four nonameric LH2 complexes from *Rhodopseudomonas palustris*(41) were investigated: three complexes with conventional B800–850 absorption and one with a B850 Q_*y*_ band so blue-shifted that it merged with the regular B800 absorption, producing a single enhanced absorption peak at 803 nm. A careful comparison showed that, within the accuracy of the structural data, the positions of the B850 bacteriochlorin rings for B800–B850 and B800–800 complexes were essentially identical, with no significant differences in Mg^2+^–Mg^2+^ distances. The only difference between the complexes is the absence of H-bonds to the 18-BChl ring of BChls in the B800–800 LH2. Thus, H-bonding appears to play a decisive role in tuning the absorption of at least some LH2 complexes.

To clarify these issues, in this research, exciton and site energy spectral shift components in the well characterized H-bond mutant bacterial light-harvesting complexes are first quantitatively assessed in comparison with those in pseudowild type (p-WT) complexes (please consult Materials and Methods sections for the distinction between WT and p-WT complexes). The fluorescence anisotropy excitation and hole-burning spectroscopies applied at 4.5 K to improve spectral resolution enabled the determination of not only the bandwidth of light-harvesting excitons but also the benchmarking of some important light-harvesting exciton model characteristics.^42^ This combination of approaches also reveals the intricate details of how the exciton and site energy shift mechanisms work in unison to achieve optimal spectral tuning.

The samples under study comprise light-harvesting 1 (LH1)–reaction center (RC)–PufX core (hereafter named as LH1-RC) and light-harvesting 2 (LH2) peripheral complexes from *Rhodobacter (Rba.) sphaeroides* with well-defined^43−46^ tertiary structure H-bonds between the C3 acetyl carbonyl groups of antennae BChl pigments and conserved tryptophan (W) and tyrosine (Y) residues of the surrounding protein. Both LH1 and LH2 appear as oligomers of basic heterodimeric structures composed of membrane-spanning α-helical α- and β-polypeptides, with each apoprotein unit noncovalently binding two (LH1) or three (LH2) BChl molecules. The number of apoprotein units and large-scale architecture of the complexes is species dependent (see refs (35 and 47) for recent reviews). In the monomeric LH1-RC-PufX complex from *Rba. sphaeroides*, the presence of PufX polypeptide breaks the continuity of the circular array of LH1 αβ oligomers,^46^ but most of the core complexes assemble into a dimeric nonplanar S-shaped array of 28 αβ-BChl_2_ units that enclose two RCs.^43,44^ In LH2, αβ oligomers form a closed nonameric ring of transmembrane apoproteins that bind nine subunits.^45^ Bacteriochlorophylls close to the cytoplasmic surface of LH2 and a ring of 18 overlapping BChls nearer to the periplasmic surface forming two rings are, respectively, named as B800 and B850 according to their Q_*y*_ exciton absorption band positions at ambient temperature. The single absorption peak of LH1 is similarly designated as B875. It is important to recognize that Q_*y*_ bands in B875 and B850 pigment structures represent not a single transition but a manifold of exciton states that, describing the assembly of either 56 (B875) or 18 (B850) closely coupled BChls, extends at cryogenic temperatures toward shorter wavelengths to about 750 nm.^42^ Basic quantum mechanical models to understand the spectroscopy of cyclic light-harvesting complexes of purple bacteria are reviewed in refs (9, 48, and 49). A full atomic resolution model of a functional chromatophore vesicle of *Rba. sphaeroides* is found in ref (50).

## 2 Materials and Methods

### 2.1 Samples

Detergent-purified LH1-RC and membranous LH2 H-bond mutant complexes from p-WT *Rba. sphaeroides* were prepared as described earlier.^37−39,51^ The term “p-WT” emphasizes that the samples with a standard set of H-bonds come from mutant species that develop either core or peripheral complexes but not both like in WT species. This difference may be vital, since recent data^52^ indicate that intracytoplasmic membranes of *Rba. sphaeroides* with selectively expressed chromoprotein complexes have different phospholipid composition. The concentrated samples were stored at −78 °C. Prior to use, the samples were diluted with 20 mM Hepes pH 7.8 buffer. In the case of purified complexes, the buffer solution contained 0.05% of a detergent *n*-dodecyl β-d-maltopyranoside to avoid aggregation. For low-temperature measurements, glycerol with a 2:1 volume ratio was added to the sample solution to obtain transparent glassy samples, while simultaneously the detergent concentration was increased to ∼0.12% to suppress aggregation. In the LH1-RC complex, one of the mutations (α-W_+11_F), replaces the residue that normally H-bonds to the C3 acetyl carbonyl group of one of the BChls in the αβ-BChl_2_ apoprotein unit, while another (β-W_+9_F) disrupts the H-bond to the C3 acetyl carbonyl of the pairing BChl within an LH1 αβ oligomer. The double mutant core complexes with replacements in both sites are structurally compromised and are unstable in detergent. For LH2, only membrane-embedded complexes are available that carry the H-bond breaking single (α-Y44F) or double (α-Y44F + α-Y45L) residue replacements.

### 2.2 Spectroscopy

The transmission (transformed to absorption), fluorescence anisotropy excitation,^53,54^ and hole-burning action spectra^55^ were recorded by a system that comprised a 0.3-m focal length spectrograph Shamrock SR-303i equipped with a thermo-electrically cooled CCD camera DV420A-OE (both Andor) and a model 3900S Ti:sapphire laser of 0.5 cm^–1^ line width pumped by a Millennia Prime solid-state laser (both Spectra Physics). In the measurements of fluorescence anisotropy excitation spectra, the vertically polarized laser beam was scanned over a proper wavelength range. The anisotropy, *r*(λ), as a function of the excitation wavelength λ is defined as *r*(λ) = (*I*_*vv*_ – *I*_*vh*_)/(*I*_*vv*_ + 2*I*_*vh*_) (*I*_*vv*_ and *I*_*vh*_ are, respectively, the emission intensities polarized parallel and perpendicular to the orientation of the electric vector of the linearly polarized excitation laser light). The fluorescence spectra were detected through a high-contrast analyzing polarizer set parallel or perpendicular to the excitation laser beam. Another correcting polarizer was fixed at 45° to the polarization direction of the analyzing polarizer. To generate comparable results, the fluorescence was selectively recorded with a 10 nm bandwidth on the red-side slope of the emission maximum.^42^ The estimated experimental uncertainty of the baseline anisotropy value is ±0.03. In the measurements at cryogenic temperature of 4.5 K, PMMA plastic cuvettes (Brand) were placed into a liquid helium bath cryostat (Utreks). Temperature was recorded with a precision of ±0.5 K by a Lakeshore Cryotronics calibrated silicon diode temperature controller Model 211.

### 2.3 Model Calculations

Absorption and fluorescence excitation anisotropy spectra were simulated by using a simple exciton model based on dipole–dipole interactions between all the BChls, treated as transition dipole moments (TDMs).^1,11,13,30^ The hole burning spectra were extracted as the absorption contour of the lowest-energy exciton state. To reproduce the spectra, it was necessary to introduce the randomness into the model.^9,15,56^ A Gaussian-shaped diagonal (site-energy) disorder was implemented, accounting for the fact that chromoprotein systems encompass two qualitatively different types of static spectral disorders:^12^ one (Γ_idis_) arising from intraprotein (or internal) and the other (Γ_edis_) from interprotein (external) variations of the parameters. Notably, only the internal disorder component is subject to exciton exchange narrowing.^57^ In the Gaussian approximation, the total disorder can be expressed as Γ_IDF_ = (Γ_idis_^2^ + Γ_edis_^2^)^1/2^. Absorption spectra were obtained by averaging over the results of 5000 diagonalizations of the dipole-Hamiltonian matrix, with each calculation generating a stick spectrum that represents a slightly different realization of site energies. The stick spectra, corresponding to exciton states, were subsequently dressed with line shape functions as described in ref (12).

The input parameters for the model included the magnitude of TDMs (μ), the site energies of the excitonically BChls (*E*_site_), structure parameters (such as the radii and TDM orientation angles for the α and β rings), the widths of internal (Γ_idis_) and external (Γ_edis_) diagonal disorders,^12^ and the dimensionless correction factor (*F*) for the two largest interactions between the nearest neighbor TDMs from dipole-dipole interaction calculations.^58^ The initial structural parameters for the BChl (TDM) arrangements were sourced from X-ray and cryo-EM studies via the RCSB protein Data Bank. While the ring radii were fixed to the given values, all other parameters were allowed to vary within their physically reasonable range during the fitting process, resulting in adjusted model parameters. To reduce the number of variable parameters no distinction was made between the site energies of α- and β-bound BChls in the αβ-BChl_2_ heterodimer units. Although this simplifying assumption ignores structural data and high-level quantum chemical calculations,^28,59^ the spectroscopic methods used in this work are sensitive only to the “average” site energies.

The primary figure of merit in the fits was the visual resemblance between the simulated and observed absorption spectra, along with the alignment of the second (high-energy) anisotropy dip, which determines the width of the exciton band. The blue side of the simulated absorption spectrum was intentionally kept “thinner” to account for a possible vibronic contribution to the measured spectrum. For isolated LH1-RC complexes, the positions and widths of the inhomogeneous distribution functions (IDFs) related to the lowest-energy (*k* = 0) light-harvesting exciton states were explicitly fitted to the observed data. However, for LH2 membrane systems, it was assumed that the measured IDFs could be additionally red-shifted by a few tens of wavenumbers due to energy transfer to the lowest energy states of the entire membrane. MATLAB (from MathWorks) was used for model calculations, and Origin 9.0 (from OriginLab) was used for measured data processing and for preparing the illustrations. A detailed description of our exciton model and its in-depth analysis will be presented in a separate publication.

## 3 Results

**Figure 1:**
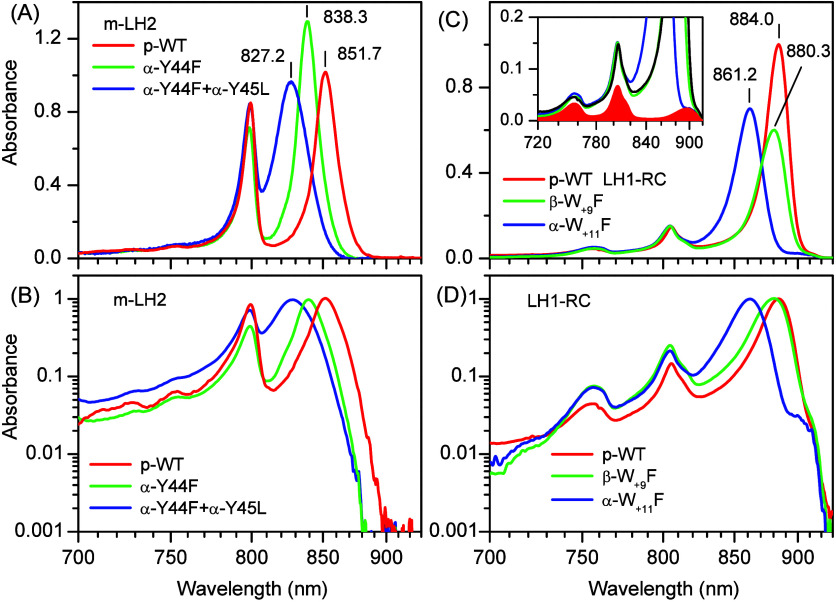
Near-infrared absorption spectra of p-WT (red line) and specific H-bond mutant LH2 (on the left, panels A, B) and LH1-RC (on the right, panels C, D) complexes (green and blue lines as indicated in legends) from *Rba. sphaeroides* recorded at 4.5 K. The spectra in panels A and B are normalized according to integral intensities of the B800 (A) or RC (C) bands, while the spectra in panels B and D plotted in logarithmic absorbance scale are normalized by the B850/B875 peak intensities to compare their shapes, simultaneously bringing up the small shape variations at the exciton band edges. All the spectra are presented on a linear-energy reciprocal wavelength scale. Numbers label the spectral positions λ_Q_ in nanometers of the Q_*y*_ exciton absorption bands. The inset of panel C shows amplified LH1-RC absorption spectra along with the underlying RC spectrum drawn in red.

Depicted in Figure 1 are near-infrared exciton absorption spectra of the studied p-WT and H-bond mutant LH2 and LH1-RC complexes. As explained in the Materials section, the term “p-WT” refers to samples from mutant species that develop either core or peripheral complexes, but not both, as seen in WT species. Given the close resemblance of the data for p-WT and WT complexes that, except for the Q_*y*_ and IDF peak positions, fall within the bounds of the experimental uncertainty,^42^ we have decided to drop this distinction for clarity in the main text further below. However, for the sake of accuracy, we will maintain the differentiation in tables and figure legends. The spectra in Figure 1 recorded at 4.5 K are specifically normalized, as described in the figure caption. The spectra of LH2 complexes (Figure 1A) expose two exciton peaks that correspond to loosely packed (i.e., weakly coupled) BChls in the B800 ring, represented by left-hand band in this figure, and tightly packed (strongly coupled) BChls in the B850 ring shown on the right. The spectra of LH1-RC complexes (Figure 1B) are dominated by the absorbance of LH1 (B875) excitons. The contribution of RCs to this spectrum (two peaks at 756 and 805 nm and a low-energy tail evident past about 900 nm) is relatively minor. According to previous knowledge summarized in ref (9) all these spectra are associated with the lowest-energy singlet electronic (Q_*y*_) transition in individual BChl molecules. A detailed account of the low-temperature spectroscopy of the native core and peripheral light-harvesting complexes from *Rba. sphaeroides* can be found in refs (60 and 61).

The B850/B875 bands of light-harvesting excitons appear relatively broad, especially considering the low experimental temperature of 4.5 K. These bands also exhibit a characteristic asymmetric shape, with a tail extending toward higher energies, as is especially well seen in the logarithmic-scale presentation of the spectra in panels B and C of Figure 1.

**Figure 2:**
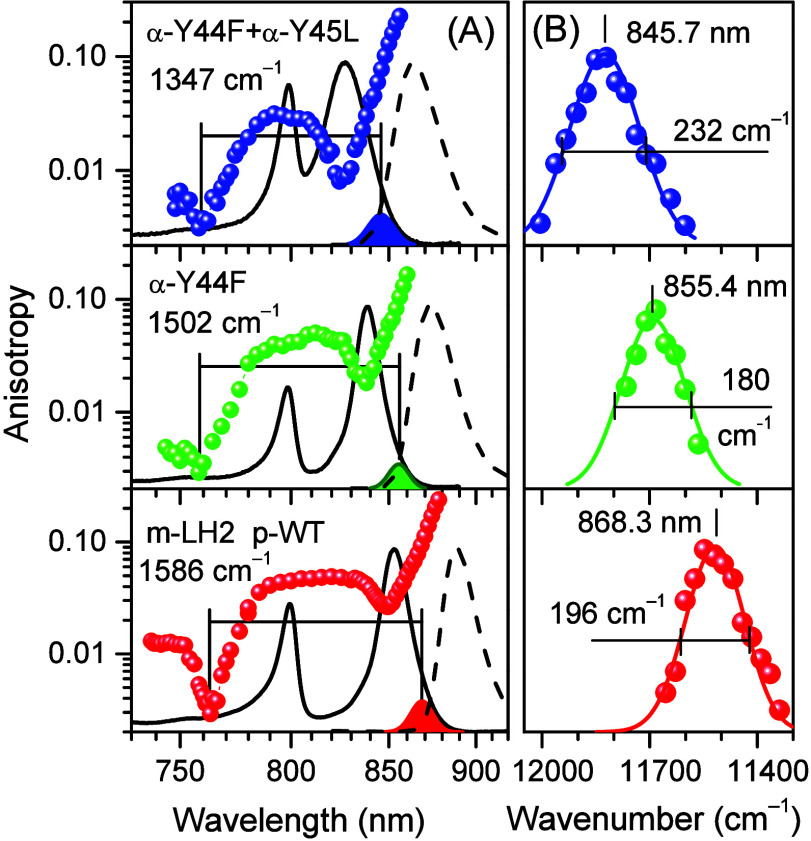
(A) Fluorescence anisotropy excitation (colored balls) and hole-burning action (color-shaded IDF shapes) spectra of p-WT (bottom spectrum) and specific H-bond mutant LH2 complexes as indicated. The spectra recorded at 4.5 K are plotted on a reciprocal wavelength scale. Lines connecting the scattered anisotropy data points are for leading the eye. Horizontal lines measure the exciton bandwidths in wavenumber units. Shown in the background are peak-normalized black solid-line absorption and dashed-line fluorescence spectra. Note that a logarithmic intensity scale is used for anisotropy to highlight the weak structures observed around 730–770 nm. All other *y*-scales are linear. (B) Expanded views of arbitrarily peak-normalized IDFs with indicated peak positions and widths, Γ_IDF_. Shown with solid lines are Gaussian approximations of the scattered experimental data.

**Figure 3:**
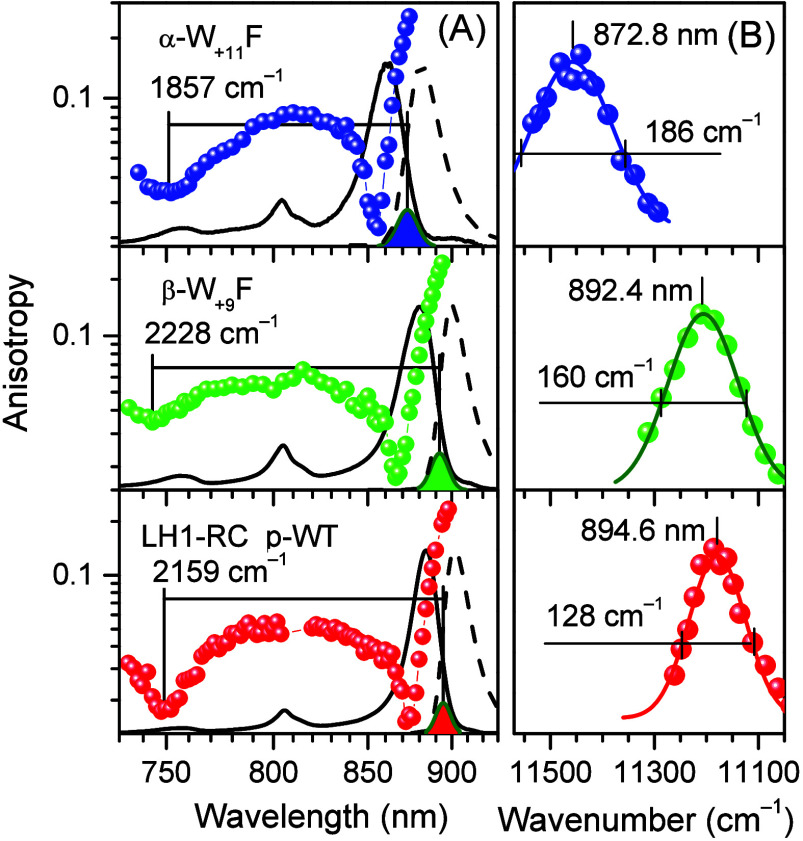
Fluorescence anisotropy excitation (A) and peak-normalized hole-burning action spectra (B) of p-WT (bottom spectrum) and specific H-bond mutant LH1-RC complexes recorded at 4.5 K. Note that a logarithmic intensity scale is used for anisotropy to highlight the weak structures observed around 730–770 nm. Other features are as for Figure 2.

To qualitatively understand these spectral features, it is important to recall that in the cyclic arrangement of BChls in model LH2 and LH1 complexes the *Q*_*y*_ transitions of 18 or 32 closely coupled BChl pigment chromophores form a manifold (or band) of 18 or 32 exciton states indexed from *k* = 0 to *k* = 9 in LH2 and from *k* = 0 to *k* = 16 in LH1. In ideal structures, only the exciton band-defining states (*k* = 0 at the bottom of the bands, and *k* = 9 and *k* = 16 at the top of the respective bands) are nondegenerate, while the rest of the states are pairwise degenerate. Due to the orientation of the transition dipole moments almost in the B850/B875 ring planes, most of the exciton dipole strength is concentrated not into the lowest-energy *k* = 0 state, but to two *k* = ±1 states higher in energy, making the greatest contribution to the observable near-infrared absorption band. The remaining states are optically dark, generally preventing the determination of the full exciton bandwidth in LH2/LH1 complexes through standard absorption measurements. However, the inherent disorder in these systems disrupts the degeneracy of the states, transferring some intensity to neighboring, previously dark exciton states (*k* = 0, *k* = ±2, *k* = ±3, etc.) at the expense of the bright *k* = ±1 states. Notably, the oscillator strength acquired by the *k* = 0 state is utilized in this work to determine the low-energy exciton band edge using hole-burning spectroscopy, as shown in Figures 2 and 3. The scheme illustrating the exciton energy levels structure in LH2 can be found, e.g., in ref (48).

Consistent with refs (37 and 38), the removal of specific H-bonds within genetically engineered complexes induces a blue shift and broadening of the B850 and B875 absorption bands compared to those in WT complexes—refer to Table 1 for quantitative details. Similar spectral responses have been observed in WT complexes perturbed by high-pressure compression that leads to disruption of H-bonds.^62,63^ Apart from shift and broadening, the normalized spectra in Figure 1 reflect an intriguing contrast in integral intensities within the B850 and B875 bands, which generally rise following disruption of H-bonds in LH2 and decrease in LH1 complexes. Since, according to ref (5), the oscillator strength of the LH2 Q_*y*_ transition is sensitive to macrocycle ring distortions, but relatively unresponsive to disturbances of H-bonds, this phenomenon may be suggestive of different macrocycle ring distortions brought about by the disruption of H-bonds in those complexes.

**Table 1:** Parameters Characterizing the B875 and B850 Exciton Absorption Spectra in Purified LH1-RC and Membrane-Embedded (m-) LH2 Complexes, Respectively, Recorded at 4.5 K^a^

Complex		λ_Q_ (nm)	Γ_Q_ (cm^–1^)	*I*_Q_ (rel. u.)	Δ*E*_ex_ (cm^–1^)	λ_IDF_ (nm)	Γ_IDF_ (cm^–1^)	Δ*E*_0_ (cm^–1^)
LH1-RC	p-WT	884.0	270	1.00	2159	894.6	128	134
	β-W_+9_F	880.3	333	0.70	2228	892.4	160	154
	α-W_+11_F	861.2	334	0.87	1857	872.8	186	155
m-LH2	p-WT	851.7	272	0.98	1586	868.3	196	224
	α-Y44F	838.3	250	1.17	1502	855.4	180	244
	α-Y44F + α-Y45L	827.2	420	1.31	1347	845.7	232	264

a Estimated experimental uncertainties of the parameters: ±0.5 nm in the case of λ_Q_ and λ_IDF_, the Q_*y*_ and IDF peak wavelengths, respectively; ±(10–20) cm^–1^ in the case of Γ_Q_ and Γ_IDF_, the full widths at half-maximum of the Q_*y*_ and IDF bands, respectively; ±10% in the case of *I*_Q_, the integral intensity of the Q_*y*_ band; ±20 cm^–1^ in the case of Δ*E*_ex_, the exciton bandwidth, calculated as explained in the text; ±10 cm^–1^ in the case of Δ*E*_0_, the energy gap between the Q_*y*_ and IDF band peaks: Δ*E*_0_ = *E*_Q_ – *E*_IDF_.

The general additivity of the spectra of WT LH1 and RC complexes described in ref (61) implies rather weak electronic coupling between them. The red tail observed in LH1-RC complexes, proven to originate from a broad absorption spectrum of the RC special pair with a maximum at 896 nm at 4.5 K, becomes even more conspicuous in the engineered LH1-RC complexes. This observation is attributed to the fact that the loss of H-bonds in LH1 has a minimal impact on the spectrum of RCs, while blue-shifting and attenuating the LH1 spectrum. Therefore, progressively larger segments of the underlying RC spectra are unveiled, as seen in Figure 1D. These featured changes remained elusive in earlier studies of genetically engineered complexes, where the spectra were recorded either at ambient temperature or at 80 K.

Upon closer examination of the data presented in Figure 1 and Table 1, it becomes apparent that the correlations between the blue shifts of the Q_*y*_ spectra, their widths, and intensities lack a discernible systematic pattern. Most strikingly, one can notice significant narrowing, instead of expected broadening, of the α-Y44F mutant spectrum of LH2. This may be a sign of either increased exciton coupling or reduced disorder, or both combined. Currently, there is no unique interpretation of the effects of H-bond disruption on B850/B875 exciton bands, and an understanding of these spectral changes minimally requires measuring their exciton bandwidths (Δ*E*_ex_) in comparison with those in WT complexes, as will be described next.

As noted earlier, the dipole strengths of the exciton states defining the exciton band edges in cyclic light-harvesting pigment aggregates are optically weak. This is due to the circular symmetry of the complexes and the near in-plane orientation of the Q_*y*_ transition dipole moment vectors of the BChl pigments. Consequently, in this work, the width of the exciton band (Δ*E*_ex_) is determined operationally as the energy difference between the high-energy dip in the anisotropy spectrum—arising from exciton states at the top of the exciton band—and the peak of the IDF, which measures the average position of the lowest-energy (*k* = 0) exciton state.

In several earlier works,^53,56,64^ a different measure was used: the energy difference between the high-energy and low-energy dips in the anisotropy spectrum, due to the absence of hole-burning data at the time. The low-energy dip in the anisotropy spectrum results from strong exciton transitions contributing to the main Q_*y*_ exciton absorption band. Therefore, the present Δ*E*_ex_ values are not directly comparable to those in earlier references. However, as discussed in ref (42), both measures respond similarly to changes in the exciton structure of cyclic light-harvesting complexes.

All the relevant experimental spectra (absorption, fluorescence, polarized fluorescence excitation, and hole-burning action) recorded at 4.5 K for LH2 and LH1-RC complexes are presented in Figures 2 and 3, respectively. In terms of exciton bandwidth, the disruption of H-bonds results in significant (up to 15% in LH2 and 14% in LH1-RC) narrowing of Δ*E*_ex_. The only exception is the β-W_+9_F mutant core complex, where Δ*E*_ex_ actually increased approximately 3% compared with the WT complex. It is also noteworthy that, again with the exclusion of the β-W_+9_F complex of LH1, the blue anisotropy dips are apparently unaffected by the alterations of H-bonding within the experimental uncertainty. This importantly suggests that the B850/B875 absorption band shifts observed in the H-bond mutant complexes relative to the band positions in respective WT complexes may mainly arise from changes in exciton bandwidth, rather than from pigment site energy variations. The outlier behavior of the β-W_+9_F mutant may then represent a rare scenario, where the site energy shift contribution into the B875 absorption band shift is relatively more significant. Quantitative analysis performed below appears to support this qualitative conclusion (see Discussion and Table 4).

One of the key properties of delocalized excitons is their ability to mitigate the effects of various static and dynamic perturbations on site energies. As a result, exciton formation is generally associated with sharper spectra compared to those of ensembles of individual sites.^57^ However, due to significant lifetime broadening (on the order of 50–100 cm^–1^)^64^ of the higher-energy exciton states on the current samples, this effect can only be studied in the slower decaying^65^ lowest-energy exciton states that contribute to the IDF band. Figures 2B and 3B (and Table 1) show that, along with a blue-shift, the disruption of H-bonds is almost always (with the exception of the α-Y44F mutant of LH2 already mentioned) accompanied by broadening of IDF. Additionally, the value of Γ_IDF_ is much greater in peripheral complexes than in core complexes, a feature that correlates well with the stronger exciton coupling (i.e., broader Δ*E*_ex_) observed in core complexes.

**Figure 4:**
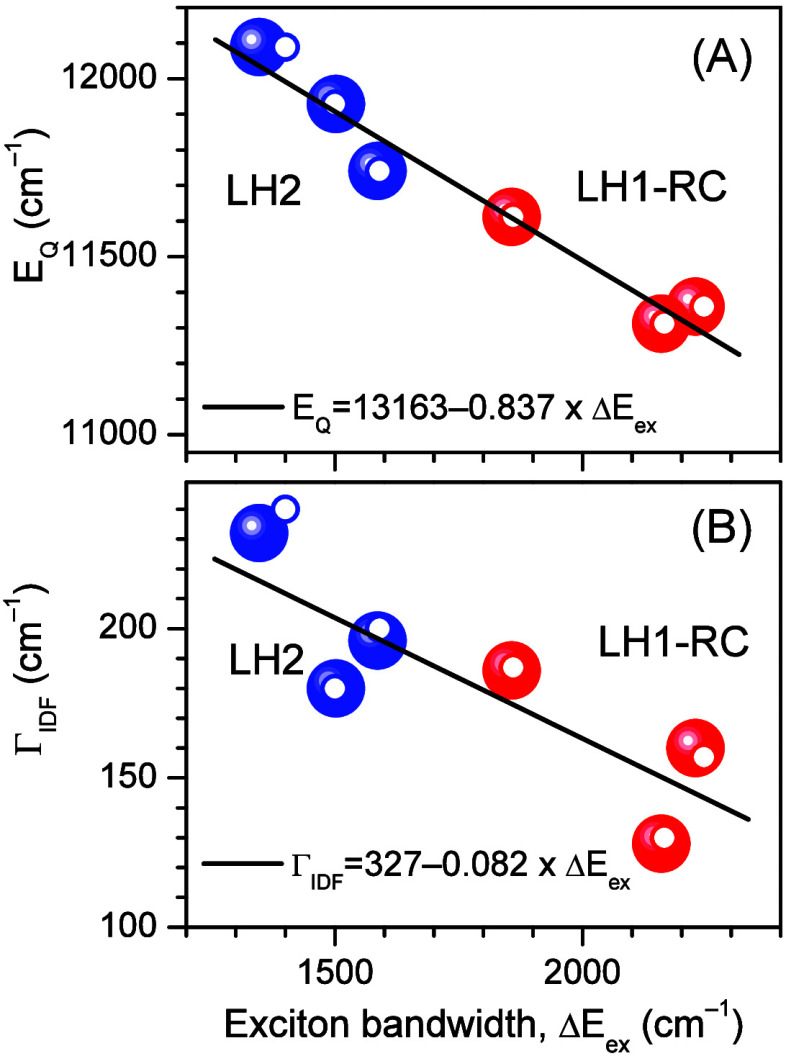
Correlations between the experimental (colored balls) and simulated (small colored rings) values of *E*_Q_ and respective exciton bandwidths Δ*E*_ex_ (A) and between the width of Γ_IDF_ and Δ*E*_ex_ (B). Blue and red symbols designate the data for LH1-RC and LH2, respectively. Lines represent linear regressions of the experimental data.

Figure 4 has been designed to illustrate the existing correlations between *E*_Q_ and Δ*E*_ex_ (Figure 4A) and between Γ_IDF_ and Δ*E*_ex_ (Figure 4B) in LH2 and LH1-RC complexes, aiming to provide a tangible physical context for these observations. Despite the fact that LH2s are membrane embedded and LH1-RCs are detergent solubilized complexes, rough linear correlations between *E*_Q_ and Δ*E*_ex_ embracing both types of complexes are observed in Figure 4A. These connections effectively capture the interplay between the red-shifting of the Q_*y*_ absorption band and the broadening of the exciton band. A converse scenario involving blue-shifting and the narrowing of Δ*E*_ex_ naturally also holds. Due to an approximate relationship that ties Δ*E*_ex_ with the nearest-neighbor exciton coupling energy, *V*, expressed as Δ*E* ≈ 4 V,^9^ a similar connection between *E*_Q_ and *V* is anticipated.

The width (Γ_IDF_) of the IDF is widely regarded in the literature as a valid measure of the energy disorder (static or dynamic) of Q_*y*_ exciton states.^66,67^Figure 4B roughly demonstrates that stronger exciton couplings generally result in finer IDF bands. This is because the degree of the exciton line narrowing is a function of the ratio Γ/*V*,^12,13^ where Γ is the width of the inhomogeneously broadened spectrum of uncoupled sites. Deviations observed from this rule (see ref (42) for representative examples) can be explained by the simultaneous presence of internal and external type of disorders, from which only the first is subject to exciton exchange narrowing.^57^

The functional dependence of Γ_IDF_ on Δ*E*_ex_ appears less robust than that of *E*_Q_ on Δ*E*_ex_. This discrepancy may be due to the fact that protein solutions frozen to cryogenic temperatures in the presence of cryoprotectors such as glycerol form glasses, whose properties generally depend on the ill-controlled sample preparation history. Based on our extensive experience, the width of the IDF is much more sensitive to this factor than the absorption band position. The relatively large experimental uncertainty assigned to Γ_IDF_ (see Table 1) quantitatively reflects this aspect. Therefore, for meaningful analyses of the data in Figure 4 and Table 1 it is important to consider the contributions of both internal and external disorder, as well as how these factors vary depending on the type of chromoprotein (LH1-RC or LH2) and the sample preparation history.

In retrospect, the selection of hole-burning spectra of the specific α-Y44F mutant LH2 sample for fitting seems not to be most representative, as the average value of Γ_IDF_ from multiple measurements is reported in ref (42) to be Γ_IDF_ = 210 cm^–1^, instead of Γ_IDF_ = 180 cm^–1^ according to Table 1 and Figure 2. However, we maintained this choice because all other measurements were performed on the same sample, as required.

Finally, it is crucial to recognize that disrupting the H-bonds does not lead to the same effects on the widths and relative positions of the lowest-energy (IDF) and the primary (Q_*y*_) exciton absorption bands. This disparity originates from a fundamental distinction in the exciton structure of these bands. In each and every cyclic light-harvesting chromoprotein, the IDF band is linked to a single exciton state, labeled as *k* = 0 in perfect cyclic structures, whereas the Q_*y*_ band encompasses contributions from multiple exciton states, albeit in the ideal system from just two: *k* = +1 and *k* = −1. Depending on the range and type of the disorder, these multiple exciton states can exhibit shifts in relation to one another, also experiencing intensity and spectral shape changes due to mutable symmetry constraints on exciton transitions.

These qualitative changes are well demonstrated in Figure 1C and 1D, where relative increase of the *k* = 0 and higher energy exciton states (*k* = ±2; ±3, etc.) with respect to the *k* = ±1 states can be seen in relationship with increasing disorder. The distinct progressions observed for the LH2 and LH1-RC complexes can be attributed to varying exciton coupling strengths and exciton state densities observed in LH2 and LH1.^49^ So, the greater density (in terms of the states per frequency unit) of exciton states in LH1 corresponds to smaller Δ*E*_0_. Conversely, the greater exciton couplings in LH1 with respect to LH2 correlate with an enhanced ability to alleviate various kinds of perturbations.

## 4 Discussion

**Figure 5:**
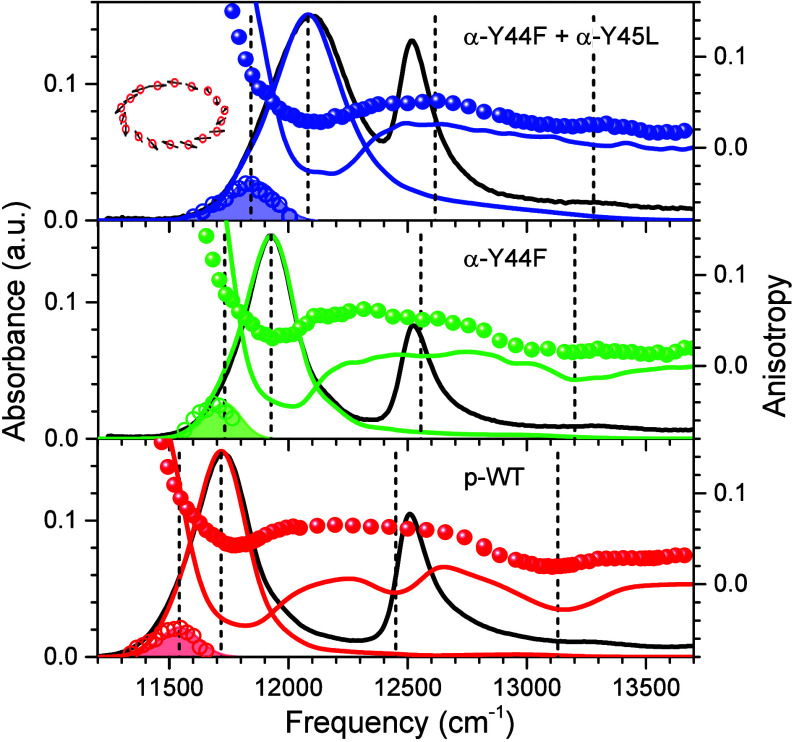
Comparison of the experimental exciton absorption (solid black line), IDF (open colored rings), and fluorescence anisotropy excitation (colored balls) spectra of WT (bottom panel) and H-bond mutant (middle and top panel) LH2 membrane complexes, as indicated, with the model spectra of the B850 light-harvesting complex. For better distinction, the model IDF spectra are shaded. Same color code as in Figure 2 is applied. Vertical dashed lines designate (counted from left to right) the calculated low-energy exciton band boundary coinciding with the IDF peak, Q_*y*_ absorption maximum (*E*_Q_), site energy (*E*_site_), and high-energy exciton band boundary, defined as the peak of the distribution of the exciton states at the top of the Q_*y*_ exciton band. The inset in the top panel shows schematic structure of the B850 ring, where 18 BChls are represented by their transition dipole moments.

**Figure 6:**
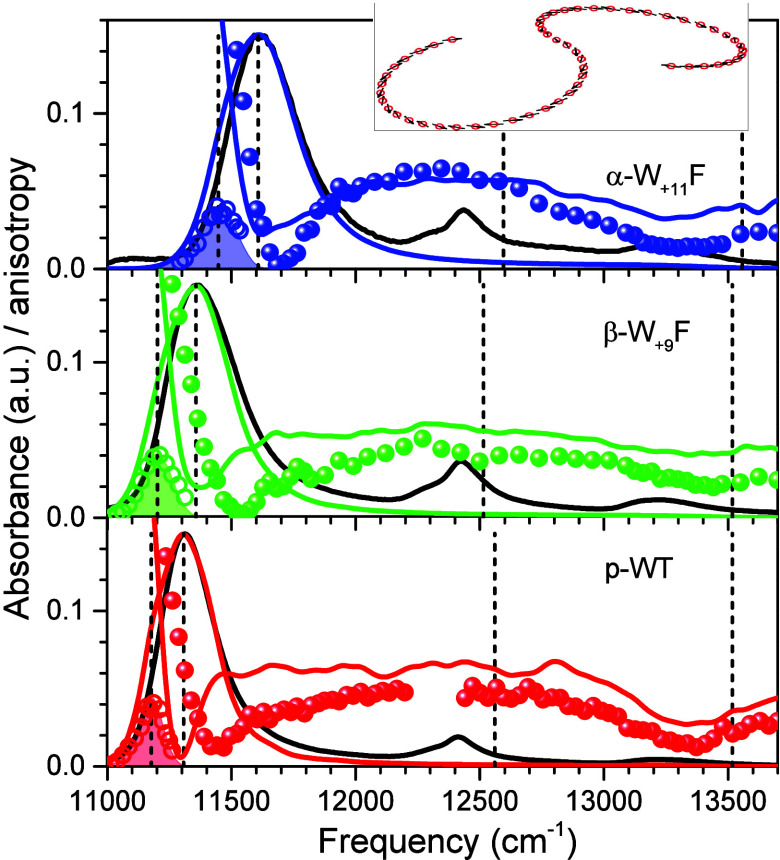
Comparison of the experimental exciton absorption (solid black line), IDF (open colored rings), and fluorescence anisotropy excitation (colored balls) spectra of WT (bottom panel) and H-bond mutant (middle and top panel) LH1-RC complexes, as indicated, with respective model spectra of the B875 light-harvesting complex. For better distinction, the model IDF spectra are shaded. The same color code as in Figure 3 is applied. Vertical dashed lines designate (counted from left to right) the calculated low-energy exciton band boundary coinciding with the IDF peak, Q_*y*_ absorption maximum, *E*_Q_, site energy (*E*_site_), and high-energy exciton band boundary, defined as the peak of the distribution of the exciton states at the top of the Q_*y*_ exciton band. The inset shows schematic S-shape array of 56 B875 BChls represented by their transition dipole moments.

**Figure 7:**
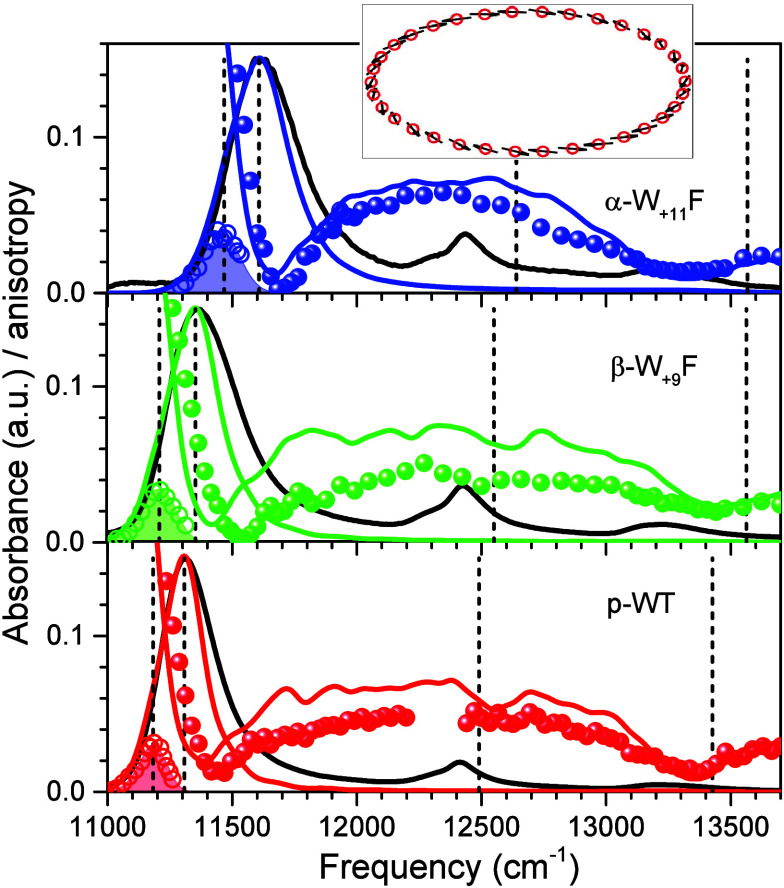
Comparison of the experimental exciton absorption (solid black line), IDF (open rings), and fluorescence anisotropy excitation (balls) spectra of WT (bottom panel) and H-bond mutant (middle and top panel) LH1-RC complexes, as indicated, with respective model spectra of the B875 light-harvesting complex. For better distinction, the model IDF spectra are shaded. Same color code as in Figure 3 is applied. The inset shows the schematic closed-ring structure of the B875 aggregate, where 32 BChls are represented by their transition dipole moments.

In this part, we simultaneously model the absorption, fluorescence excitation anisotropy, and hole-burning spectra in the B850 and B875 assemblies of BChls in LH2 (Figure 5 and Table 2) and LH1-RC (Figures 6 and 7 and Table 3) complexes, respectively, in a hope to quantify the observed spectral changes following the H-bond tailoring in terms of the site energy and exciton shift contributions. The model used is briefly described in the Materials and Methods section.

**Table 2:** Selected Model Parameters Characterizing the B850 Exciton Absorption, Hole Burning, and Polarized Fluorescence Excitation Spectra of WT and H-Bond Mutant LH2 Membrane Complexes According to Figure 5

Parameter (unit)	p-WT	α-Y44F	α-Y44F + α-Y45L
*μ* (D)	8.2	8.2	8.2
*E*_site_ (cm^–1^)	12450	12555	12615
Γ_idis_ (cm^–1^)	469	553	683
*F*	0.55	0.38	0.23
*V* (cm^–1^)	378	271	176
Γ_idis_/V	1.24	2.04	3.88
*E*_IDF_ (cm^–1^)	11542	11732	11842
Γ_IDF_ (cm^–1^)	200	180	240
Δ*E*_0_ (cm^–1^)	175	195	240
Δ*E*_ex_ (cm^–1^)	1590	1500	1400

The essential fit variables of the model as presented in Tables 2 and 3 are μ, *E*_site_, Γ_idis_, and *F*. The remaining rows designate derived parameters. Note that *V* represents the maximum exciton coupling energy after applying the correction. Additionally, recall that Γ_IDF_ is a composite parameter approximated by Γ_IDF_ = (Γ_idis_^2^ + Γ_edis_^2^)^1/2^. Only the Γ_idis_ component is crucial for constructing the exciton band structure, specifically in acquiring optical strength for the *k* = 0 band. Γ_edis_, on the other hand, simply contributes to uniform broadening of the spectra. Since the *k* = 0 (or IDF) band largely determines the low energy slope of the absorption spectrum, the Γ_idis_ was determined by best fit between the experimental and calculated absorption spectra. According to this procedure, the effect of external disorder—141 cm^–1^ in WT and to 47 cm^–1^ in both mutant complexes—was found to be relatively minor, consistent with ref (12).

**Table 3:** Selected Model Parameters Characterizing the B875 Exciton Absorption, Hole Burning, and Polarized Fluorescence Excitation Spectra of WT and H-Bond Mutant LH1 Complexes According to Figure 7

Parameter (unit)	p-WT	β-W_+9_F	α-W_+11_F
μ (D)	8.7	8.7	8.7
*E*_site_ (cm^–1^)	12490	12550	12640
Γ_idis_ (cm^–1^)	226	273	303
*F*	0.49	0.49	0.38
*V* (cm^–1^)	484	484	375
Γ_idis_/V	0.47	0.56	0.81
*E*_IDF_ (cm^–1^)	11183	11208	11468
Γ_IDF_ (cm^–1^)	130	159	187
Δ*E*_0_ (cm^–1^)	125	145	140
Δ*E*_ex_ (cm^–1^)	2165	2245	1860

In the case of LH2, reasonably good solutions depicted in Figure 5 were obtained assuming a closed-ring structure of the B850 aggregate. However, the calculated IDF band positions are systematically shifted more toward the blue compared to experimental bands. Additionally, the calculated anisotropy spectra show deeper dips than the experimental ones, with their red dips also consistently blue-shifted relative to the experimental data. These discrepancies, previously noted in ref (69), can be attributed to two primary reasons.

First, the excitation energy transfer processes present in the experimental network of LH2s in chromatophore samples are absent in the model B850 samples representing isolated LH2 complexes. This mostly affects the shape and position of the low-energy dip in the anisotropy spectrum. Second, the model calculations lack phonon and vibronic effects, which influence both the high-energy side of the absorption spectrum, as explained, e.g., by Ahad et al.,^21^ and the position of the low-energy anisotropy dip. The latter finding is a fresh result yet to be analyzed.

There are also important experimental factors that may contribute to the observed differences. One is the hole-burning action spectrum artifact, which effectively increases the experimental Δ*E*_0_ values due to spectral overlap between the IDF and distributions of higher energy exciton states. According to our estimates, this correction may be in the range of 20–30 cm^–1^. Another factor is the baseline uncertainty in anisotropy measurements, which, as indicated in the Materials and Methods section, may reach ±0.03.

The significant dependence observed for the scaling factor *F* is noteworthy. In our simulations, we chose to fix the transition dipole moments at a reasonable value while varying the dipole orientation angles and the scaling factor. This approach was adopted because (i) according to X-ray structural data,^68^ the absence of H-bonds between the acetyl carbonyls of BChls and the surrounding protein permits changes in geometry and, consequently, exciton coupling, both within the αβ-BChl_2_ heterodimeric units and between them, and (ii) the loosened acetyl carbonyls can freely adjust their conformation relative to the BChl macrocycle plain, which sensitively alters the BChl site energies.^28^ The dynamic changes also contribute to spectral broadening. However, the observed strong variation in *F* might be mitigated by making additional adjustments to the transition dipole moment.

The changes in the best-fit parameters of *E*_site_ and Δ_ex_ upon mutation of LH2 complexes, relative to WT species, are provided in Table 4. These data suggest that the site energy and exciton shift mechanisms are equally instrumental in the blue-shifting of the Q_*y*_ exciton absorption bands in mutant complexes, in both mutants working together to enhance the effect. Quantitatively, the site-energy shift contribution appears to be greater in the single mutant, while in the double mutant, the balance leans more toward the exciton shift mechanism.

**Table 4:** Exciton Model Parameters in Wavenumber Units Characterizing the B850/B875 Band Shifts in LH2/LH1-RC Complexes, Respectively

Complex		Δ_site_ (cm^–1^)	ΔΔ*E*_ex_ (cm^–1^)
LH1-RC	p-WT	0	0
	β-W_+9_F	+60	+80
	α-W_+11_F	+150	–305
m-LH2	p-WT	0	0
	α-Y44F	+105	–90
	α-Y44F + α-Y45L	+165	–190

Unfortunately, the uniqueness of the solutions in LH2 complexes is compromised by the weakness and indistinctness of the high-energy anisotropy spectra, leaving room for alternative interpretations. Figure 5 quite clearly displays two anisotropy dips, also known from our previous studies (see, e.g., ref (69)). While membrane embedding makes the anisotropy structures less distinct, the origin of the double structure remains unclear at this time. One possible explanation could be sample heterogeneity, similar to that observed in ref (44) in some species of *Rba. sphaeroides* core complexes, though this has yet to be proven for peripheral LH2 complexes.

Despite these complications and uncertainties, our main conclusions well align with the findings of Cardosa Ramos et al.^28^ regarding the spectral modifications observed in LH2 complexes from *Rhodoblastus acidophilus* grown under high-light and low-light conditions. Their results also indicate that only a combination of site energy shift and change in electronic couplings due to rearrangements in the H-bond network can fully account for the observed spectral differences.

The results of similar analyses for LH1-RC samples are shown in Figures 6 and 7. In the former case, a nonplanar S-shape assembly of 56 B875 BChls, typical for the PufX-containing *Rba. sphaeroides* species,^43,44^ was assumed. Inspection of the data in Figure 6 reveals two qualitative inconsistencies between the experimental and simulated spectra regarding the shape of the Q_*y*_ absorption spectrum and the position of the low-energy anisotropy dip relative to the Q_*y*_ absorption maximum.

First, the calculated absorbance on the low-energy side is consistently much stronger compared to the experimental one. The high-energy side deficiency is ignored here because, as noted above, it can be readily explained by the absence of phonon and vibronic effects, along with the missing contribution of the special pair of RC in model calculations. Second, while the calculated low-energy anisotropy dip coincides with the Q_*y*_ absorption maximum, in the experimental spectra, it is always right-shifted (toward high energies) relative to the absorption peak.

Both these qualitative irregularities can be alleviated by assuming a closed-ring structure of the B875 array complete with 32 BChls, characteristic to LH1-RC complexes that miss PufX.^46^ These generally more satisfactory results are quantified in Figure 7 and Table 3. Indeed, the red sides of the calculated and experimental spectra now closely overlap, and the calculated and experimental anisotropy dips are on the right (i.e., on high-energy) side of the absorption peak. Both these alleviating effects can be explained by increased symmetry of the LH1 structure. The dependence of exciton state intensities on symmetry constraints in LH1 and LH2 complexes was demonstrated earlier.^70,71^ The present analysis thus unexpectedly suggests that LH1 protein complexes from p-WT and WT species of *Rba. sphaeroides* may have fundamentally different spatial structures. This might indicate deficiencies in the model or reflect the generally heterogeneous properties of the LH1-RC supercomplexes from *Rba. sphaeroides*.^44^

As can be seen in Table 4, the site energy and exciton shift contributions to the Q_*y*_ absorption band shits in LH1-RC complexes are similarly significant to those in LH2 complexes. However, in LH1-RC, these two contributions can either work together to enhance the blue-shifting effect, as seen in the α-W_+11_F mutant, or counteract each other, compensating for the effects caused by individual shift mechanisms, as observed in the β-W_+9_F mutant. In the α-W_+11_F mutant, the exciton shift mechanism clearly dominates in the blue-shifting of the Q_*y*_ exciton absorption band.

Our previous work^42^ additionally suggested that states at the top of exciton band respond only to relatively large structural changes. Representative examples include LH2 samples from *Rhodoblastus acidophilus* grown under high-light versus low-light conditions^72^ or prepared according to different procedures.^73^ Given the relatively larger shift of the blue side anisotropy dip in the β-W_+9_F mutant of LH1-RC observed in Figure 3, one might, therefore, conclude that the replacement of the residue at the β_+9_ position causes a greater amount of structural rearrangements in LH1 than the replacement at the α_+11_ position. Another previous study based on analyses of Raman spectra^39^ reached similar conclusion. However, this is not the case according to the data in Figure 7 and Table 4.

## 5 Summary and Concluding Remarks

How the protein environment tunes the spectra of light-harvesting pigment chromophores has long been a topic of intense study. This work elaborates on the H-bonding mechanism, an indispensable component responsible for the remarkable spectral adaptability of all phototrophic organisms.

The data obtained on light-harvesting chromoproteins from *Rba. sphaeroides* suggests that the blue shifts of Q_*y*_ exciton absorption bands observed in the H-bond mutant complexes relative to their positions in WT complexes are contributed from alterations in both exciton interactions and in pigment site energies that result the disruption of tertiary structure H-bonds. These data correct and quantify the previous prevailing belief that the spectral changes almost directly follow shifting site energies. We further show that in line with the commonly ascribed stabilizing function of H-bonds, their disruption consistently induces a substantial increase in disorder, evident from the considerable widening of the Q_*y*_ and IDF bands. The striking broadening of the IDF band, nearing almost 60% in mutant samples, can be partly attributed to the increased dispersion of acetyl carbonyl orientations relative to the BChl macrocycle ring plane upon the removal of H-bonds and partly to the constriction of the exciton bandwidth, which reduces the impact of exchange narrowing effect.^57^ It has been noted previously^42,69^ that for optimal transport of excitons toward RCs a careful tuning is required of the spectra of light-harvesting complexes with respect to each other. As can be seen from Figures 5–7, the exciton state manifolds in core and peripheral complexes perfectly match each other, whereby the high-energy edges of the exciton state manifolds in LH1 and LH2 nearly coincide. Revealing these intrinsic adaptive mechanisms not only enriches our comprehension of fundamental biological processes but also lays a promising foundation for innovative approaches in harnessing solar energy more efficiently.

Despite significant strides in understanding the responses of light-harvesting chromoprotein spectra to alterations in their tertiary structure H-bonds, several intriguing observations require further clarification. One such observation is the conspicuous opposite change in the integral intensities within the B850 and B875 bands, as shown in Figure 1 and Table 1. If accurate (there is some ambiguity in the literature regarding the relative intensity of the B800 and B850 bands,^74^ which could potentially impact the normalization process used for LH2 complexes), this may suggest that disruption of H-bonds in peripheral and core complexes leads to different macrocycle ring distortions. Another issue is the paradoxical conclusion about the different structures of LH1 complexes in WT and p-WT core complexes. A direct way to resolve this conundrum is to obtain the so far missing near atomic resolution structures of the latter supercomplexes. Furthermore, current model improvements toward explicit accounting for the electron-vibrational and electron–phonon coupling are necessary as well as feasible in the future research.

As for how literally the specific fit parameters for each spectrum should be interpreted, this is a nuanced issue. Based on our experience, it is challenging to provide precise error bounds on the fitting parameters. Being derived from rigorous modeling, they are inherently subject to the uncertainties of the experimental data and the assumptions of the model. Thus, although the obtained parameters offer valuable insights, they should be considered as estimates rather than absolute values. We are currently addressing these issues by implementing a sensitivity analysis of our model.
